# DFT Quantum-Chemical Calculation of Thermodynamic Parameters and DSC Measurement of Thermostability of Novel Benzofuroxan Derivatives Containing Triazidoisobutyl Fragments

**DOI:** 10.3390/ijms23031471

**Published:** 2022-01-27

**Authors:** Elena Chugunova, Timur Shaekhov, Ayrat Khamatgalimov, Vladimir Gorshkov, Alexander Burilov

**Affiliations:** 1 Arbuzov Institute of Organic and Physical Chemistry, FRC Kazan Scientific Center, Russian Academy of Sciences, Akad. Arbuzov str. 8, 420088 Kazan, Russia; ayrat_kh@iopc.ru (A.K.); burilov@iopc.ru (A.B.); 2 State Research Institute of Chemical Products Federal State Enterprise, Svetlaya str. 1, 420033 Kazan, Russia; timur-shaekhov@mail.ru; 3 Laboratory of Plant Infectious Diseases, FRC Kazan Scientific Center, Russian Academy of Sciences, Lobachevskogo str. 2/31, 420111 Kazan, Russia; gvy84@mail.ru

**Keywords:** benzofuroxan, triazidoisobutyl fragment, CL-14, DSC, heats of formation

## Abstract

New derivatives of benzofuroxan containing triazidoisobutyl fragments, opening the way for the creation of highly effective compositions with an increased value of energy characteristics, were synthesized for the first time. Such compounds are also an excellent platform for further modification and for the preparation of new biologically-active compounds containing tetrazole and triazole fragments. Calculations of heats of formation performed with the DFT (density functional theory) method showed that the studied compounds are high-energetic density ones, the enthalpies of formation of which are comparable to the enthalpies of formation of similar benzofuroxan derivatives and exceeds experimental enthalpy of formation of CL-14 (5,7-diamino-4,6-dinitrobenzofuroxan). The analysis of DSC indicates a sufficiently high thermal stability of the synthesized azidobenzofuroxans, which are acceptable for their use as components in the creation of highly efficient compositions with an increased value of energy characteristics.

## 1. Introduction

Currently, there is a significant increase in the interest of compounds that can be used as so-called “dual-use goods” for both civilian and military purposes. Examples of such compounds that are successfully used both in medicine and as explosives are: nitroglycerin—widely used in the manufacture of gunpowder and as a vasodilator for the treatment of diseases such as angina pectoris; pentaerythritol nitrate—which is used both in medicine for chronic coronary insufficiency and angina pectoris, and for the manufacture of capsule detonators and a (detonating) fuse-cord, etc. There are many examples of such dual-use goods.

Compounds containing the furoxan ring are dual-use compounds, exhibiting not only a wide range of different types of biological activity, such as anti-tuberculosis [1], anti-tumor [2], anti-inflammatory [3], anti-aggregant [4], etc. but can also be used as high-energy-density materials [5,6]. 

High enthalpy and increased density provide nitro derivatives of benzofuroxan with good energy characteristics [7]. Among the benzofuroxan compounds, a number of substances with high power characteristics were obtained, allowing them to be used as individual explosives, as well as components of explosives and propellants. Currently, among the industrially-developed benzofuroxans, there is only benzotrifuroxan (hexanitrosobenzene, benzotris(1,2,5-oxadiazole-2-oxide), BTF, **1a**), which is used in some types of detonators in mixtures with other explosives (for example, with HMX and 1,3,5-triamino-2,4,6-trinitrobenzene (TATB)) and with plasticizers, as well as in some solid rocket fuels [8,9,10,11]. The rest of the compounds are being actively studied and developed on a pilot and semi-industrial scale. 

Nitrobenzofuroxans are of particular interest; they combine a sufficiently low sensitivity to impact, friction, spark with increased power and thermal stability, allowing them to be used as individual explosives, as well as components of high-energy condensed systems.

**Figure 1:**
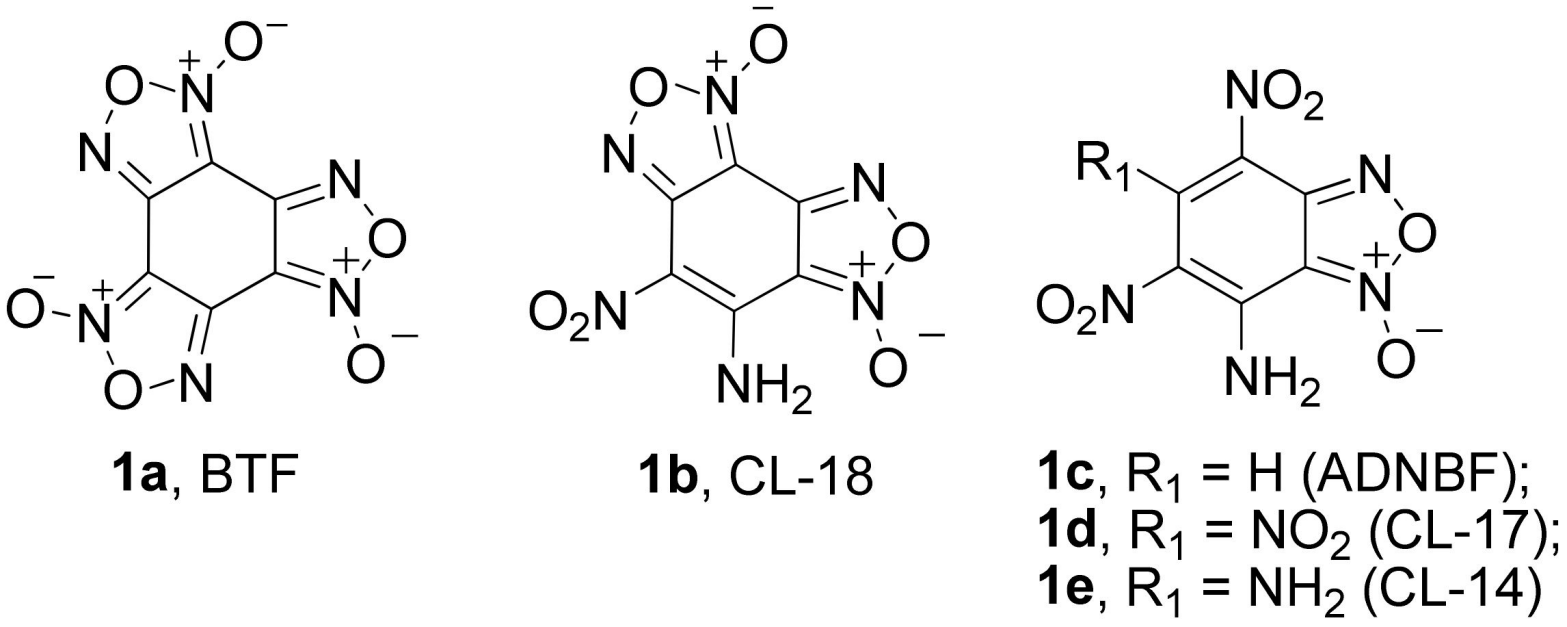
Reduced sensitivity explosives on the base of benzofuroxan.

Aminonitrobenzodifuroxan (CL-18, **1b**, Figure 1) [12], 7-amino-4,6-dinitrobenzofuroxan (ADNBF, **1c**) [13,14], 7-amino-4,5,6-trinitrobenzofuroxan (**1d**) [15], 5,7-diamino-4,6-dinitrobenzofuroxan (CL-14, DADNBF, **1e**) [16,17,18] have the highest density in the series of dinitrobenzofuroxan structures. The main disadvantages of all the described compounds are insufficiently high energy parameters.

A further increase in the energy characteristics of 5,7-diamino-4,6-dinitrobenzofuroxan can be achieved by introducing azide groups into dinitrobenzofuroxan molecules. From this point of view, *tert*-substituted derivatives of triazidoisobutane are promising fragments for introduction into benzofuroxan molecules, since the introduction of only one triazidoisobutyl fragment into the structure of the molecule will lead to an increase in the enthalpy of formation by 225.2 kcal/mol; the value is obtained as a result of the calculation for group contributions using Equation (1) [19] as follows:

−C(CH_2_N_3_)_3_ = C[+3.5] + (CH_2_)_3_[3 × (−6.1)] + (N_3_)_3_[3 × 80.0)] = 225.2 kcal/mol × 4.18 = 1080.96 kJ/mol (1)

The introduction of azide fragments is accompanied not only by high energy characteristics but also by a high combustion rate, which makes it possible to regulate the combustion rate of compositions based on them in a wide range. Currently, there are no works in the scientific and technical literature on the synthesis of azide-containing dinitrobenzofuroxans. To obtain such compounds, we selected 5,7-dichloro-4,6-dinitro- and 7-chloro-4,6-dinitrobenzofuroxans containing one or two chlorine atoms in their structures, which can be easily replaced by triazidoisobutyl fragments. 5,7-dichloro-4,6-dinitro- and 7-chloro-4,6-dinitrobenzofuroxans are convenient platforms for their further modification in order to obtain new derivatives containing various functional groups [20,21,22,23,24,25]. In this paper, we discovered new benzofuroxan derivatives containing triazidoisobutyl fragments, studies of temperature characteristics, assessment of compatibility with cellulose nitrates and quantum-chemical calculations of the thermodynamic and explosive characteristics of the obtained compounds. At the same time, the presence of azide groups in these compounds opens the way for the creation of biologically active tetrazoles and triazoles on their basis [26,27,28,29,30].

## 2. Results and Discussion

### 2.1. Synthesis of Azidobenzofuroxans

**Scheme 1:**
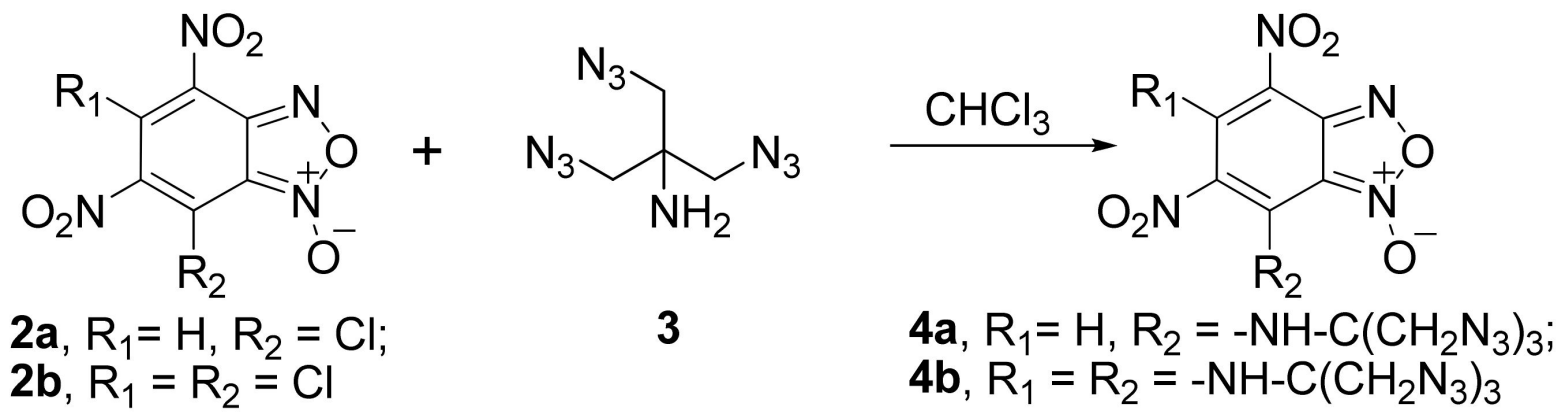
Synthesis of azidobenzofuroxans 4a,b.

Key compounds were obtained via aromatic nucleophilic substitution reaction of 7-chloro-4,6-dinitrobenzofuroxan **2a** and 5,7-dichloro-4,6-dinitrobenzofuroxan **2b** with 1,3-diazido-2-(azidomethyl)propan-2-amine **3** as a nucleophile (Scheme 1). A second equivalent of the amine was used to bind the liberated hydrogen chloride. Compounds were fully characterized through ^1^H and ^13^C NMR spectroscopy and elemental analysis.

It should be noted that the synthesized compounds **4a,b** dissolve well in ethyl acetate, alcohol–ether solvent, and diethylene glycol monoethyl ether (ethylcarbitol), which undoubtedly gives them unique properties and simplifies their introduction into spherical propellants, as well as into propellants obtained as in standard alcohol–ether technology, and technology using a hardly volatile solvent—ethylcarbitol. The lack of water solubility makes them technologically advanced in the formation of powder masses using various technologies [31,32].

### 2.2. Heats of Formation

**Figure 2:**
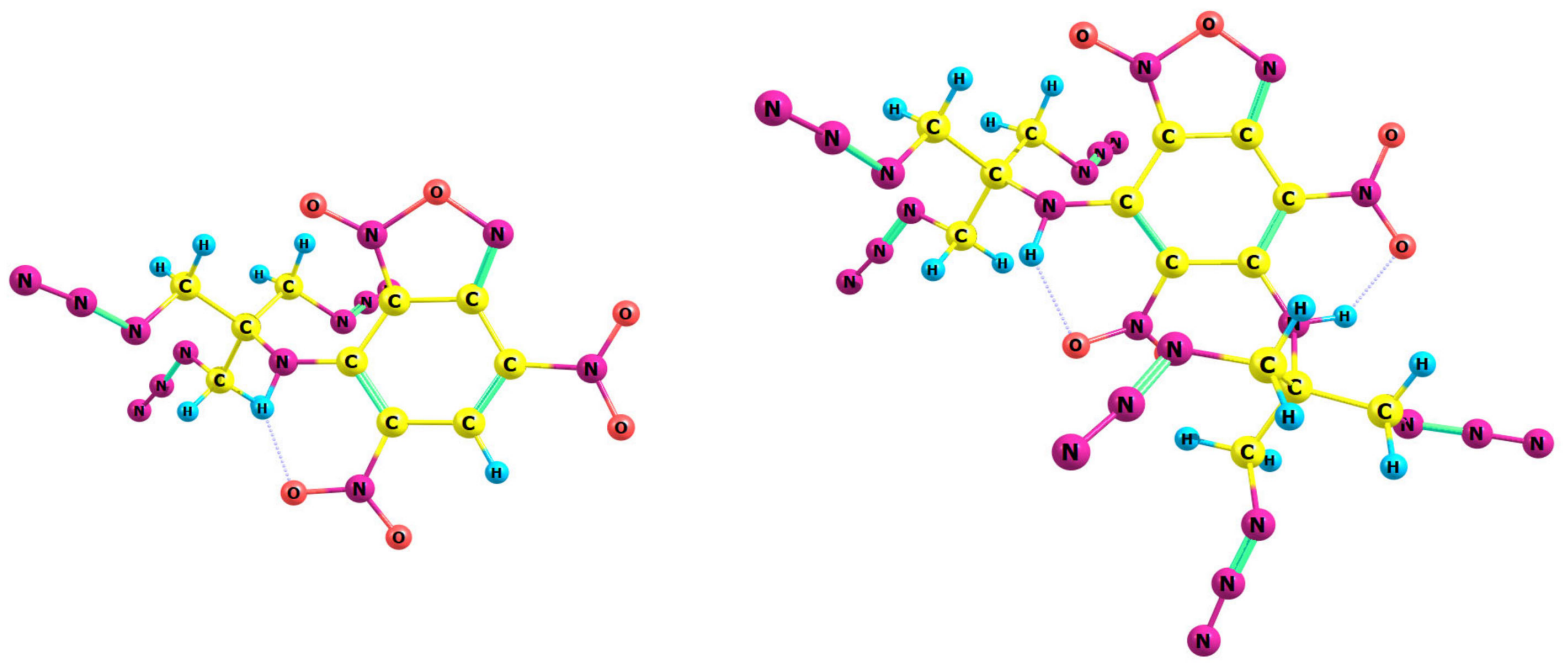
Optimized structures of compounds **4a,b**.

Calculations were performed with the Gaussian16 (Revision B.01) suite of programs [33]. The molecular structures of the investigated compounds were fully optimized using B3LYP hybrid functional [34,35] with 6-311++G(d,p) basis set with polarization and diffuse functions. Previously, this method was successfully used to describe the high energy density compounds [36,37,38]. The optimized structures of compounds **4a,b** are presented in Figure 2. As we can see from the figure, the increase in triazidoisobutyl fragments does not lead to a significant change in the structure of the benzofuroxan framework itself. As is the case with one triazidoisobutyl fragment, the addition of another one leads to the formation of a hydrogen bond with the neighboring NO_2_-group.

The gas-phase enthalpies of formation were calculated using the method of isodesmic reactions. The enthalpy of reaction is obtained by combining the DFT energy difference for the reaction. The gas-phase enthalpies of formation of reactants are calculated by using general Equation (2) for most nitroaromatic and benzofuroxan-based energetic compounds with general formula C_a_H_b_N_c_O_d_ [39].

ΔH_f_^θ^(c) = 32.76a − 33.96b + 69.12c − 116.32d + 124.8n_NO₂_ − 65.10n_Ar-NH_ − 93.64n_OH_ − 202.3n_COOH_ + 13.56(n_Ar_ − 1) + 121.4n_-N=N-_ + 223.2n_cyclo-N-O-N_, (2)

The solid-state enthalpy of formation for neutral compounds can be estimated by subtracting the heats of sublimation from gas-phase heats of formation [37,38,40]. The heat of sublimation can be estimated with Trouton’s rule [41] according to Equation (3), where T represents either the melting point or the decomposition temperature when no melting occurs prior to decomposition [37,40]:

ΔH_sub_ ≈ 188 × T_m_/[molar mass], kJ/kg, (3)

Table 1 presents the data of quantum-chemical calculations for compounds **4a,b**.

The obtained values of the heats of formation of the investigated compounds turned out to be close for those calculated by the method of isodesmic reactions and by the general Equation (2) [39]. The results of quantum-chemical calculations showed that the studied compounds have increased values of the enthalpy of formation comparable to the enthalpies of formation of similar benzofuroxan derivatives (see, for example, [37]), and in the case of 5,7-bis(1,3-diazido-2-(azidomethyl)propan-2-ylamino)-4,6-dinitrobenzofuroxan **4b**, they are almost two times higher, which is associated with an increase in the number of triazidoisobutyl fragments. For example, the 5,7-diamino-4,6-dinitrobenzofuroxan **1e** used today has an experimental enthalpy of formation equal to 337.05 kJ/kg, which is significantly lower than the benzofuroxan derivatives synthesized in this work.

### 2.3. Thermostability of Obtained Compound: TG/DSC Measurement

The temperature characteristics of azidobenzofuroxans were determined by differential scanning calorimetry (DSC) together with thermogravimetry (TG) (Table 2).

**Figure 3:**
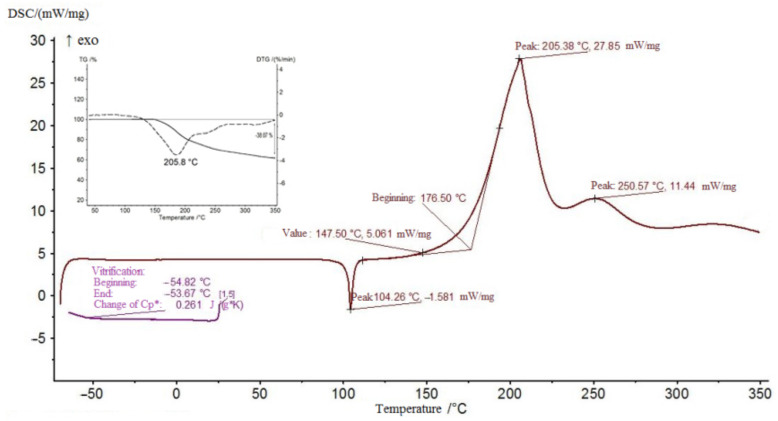
DSC curve with TG (inset) of compound **4a**.

**Figure 4:**
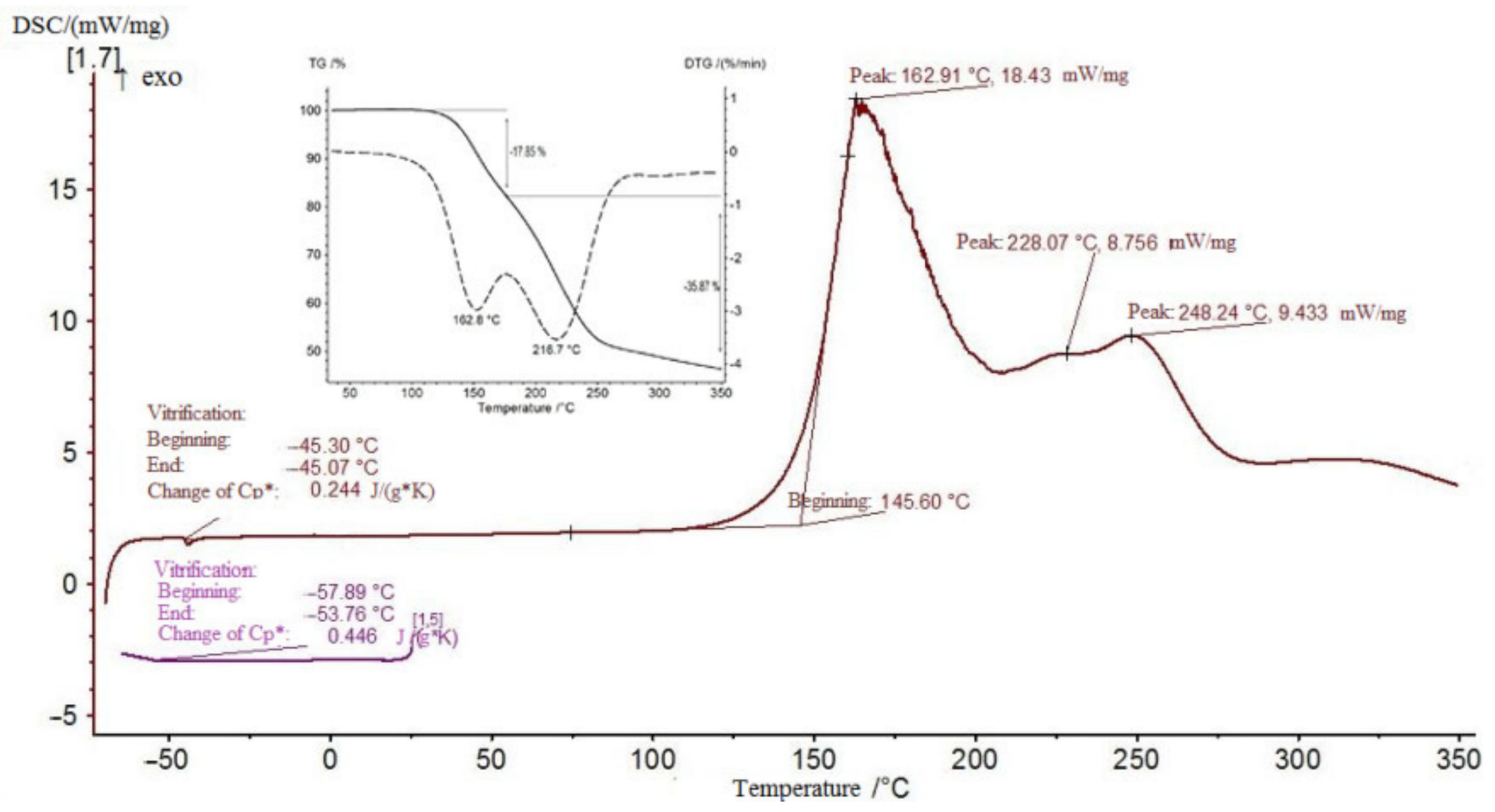
DSC curve with TG (inset) of compound **4b**.

Figure 3 and Figure 4 show the TG/DSC curves of azidobenzofuroxans. The investigated azidobenzofuroxans exhibit a similar character of mass loss; there are mass loss steps corresponding to their decomposition only. However, for **4a**, the mass loss occurs in one step (at 205.8 °C with a loss of 38.1%), while for **4b**, the mass loss occurs in two steps (at 162.8 and 216.7 °C with a loss of 17.9% and 35.9%, respectively) (see inset in Figure 3 and Figure 4). The analysis of the graphs indicates a sufficiently high thermal stability of the synthesized azidobenzofuroxans, which are acceptable for their use as components in the creation of highly efficient compositions with an increased value of energy characteristics. A comparison of the temperature characteristics of azidobenzofuroxans **4a,b** was performed with ADNBF (**1c**) and CL-14 (**1e**). It was found that the introduction of a triazidoisobutyl fragment into the structure of 7-amino-4,6-dinitrobenzofuroxan (ADNBF, **1c**) significantly shifts the decomposition temperature from 279.6 °C to 205.4 °C (Figure 3). This agrees well with temperatures of decomposition on the TG curve and the first derivative of TG (inset in Figure 3).

In addition, on the DSC curve, we observe an endo-peak at 104.3 °C, corresponding to the melting point and glass transition temperature at −54.8 °C. The introduction of two triazidoisobutyl fragments into the CL-14 structure further lowers the decomposition temperature from 289.0 °C to 162.9 °C (Figure 4). Similarly to compound **4a**, a second-order phase transition is observed on the DSC curve of the disubstituted azido derivative **4b** at a temperature of −57.9 °C, corresponding to the glass transition temperature. When compound 4b is heated from −70 °C, the second-order phase transition begins at a temperature of −45.3 °C (devitrification temperature). The DSC curves of azidobenzofuroxans **4a,b** (Figure 3 and Figure 4) show exo-peaks in the region of 228–250 °C, which probably indicate the formation of secondary structures associated with the Boulton–Katritsky rearrangement or destruction of the azide group. Table 2 shows the temperature characteristics of compounds **4a,b**, ADNBF and CL-14.

### 2.4. Thermodynamic Parameters Calculation Using REAL Version 3.0

In order to assess the possibility of using azidobenzofuroxans **4a,b** in powder compositions, their main thermodynamic characteristics (specific energy (force), potential, temperature of the combustion gases, covolume) were calculated. The calculation of the thermodynamic characteristics of benzofuroxan derivatives **4a,b** was carried out using the REAL version 3.0 computer program at a pressure of 280 MPa [44]. A comparison of the thermodynamic characteristics of azidobenzofuroxans was carried out with the currently used 5,7-diamino-4,6-dinitrobenzofuroxan **1e**. Table 3 shows the main thermodynamic characteristics of compounds **4a,b** and 5,7-diamino-4,6-dinitrobenzofuroxan **1e**.

Table 3 shows that due to the lower average molecular weight of combustion products, the adiabatic index (k = Cp/Cv) decreases in the series CL-14 > **4a** > **4b**, which, in turn, increases the potential (PP) of the compound, while the temperature of the combustion products remains at the level of 3000 K, which, in turn, indicate a low acceleration–erosion effect. 

Table 4 shows the gas composition of combustion products calculated using the program REAL version 3.0. Based on the calculation, the combustion products of components CL-14 and azidobenzofuroxans **4a,b** contain 90% of nitrogen, hydrogen and carbon monoxide. Azidobenzofuroxans **4a,b**, due to the presence of triazidoisobutyl fragments in the structure, generate a large volume of gas (N_2_), which has a positive effect on the performance of the propellant and the temperature of combustion products, in comparison with carbon monoxide (II). In the analysis of combustion products, the content of hydrogen molecules also plays an important role, which has more than an order of magnitude higher specific heat values compared to nitrogen and carbon monoxide. The content of hydrogen molecules in the compared components increases in the series CL-14 < **4a** < **4b**.

Thus, the raising of the potential (PP) of the compounds **4a** and **4b** calculated using REAL is in agreement with the results of quantum-chemical calculations and DSC measurement showing the increase in the enthalpies of formation and decomposition, respectively, of these energy-saturated products.

### 2.5. Explosive Characteristics Calculation

The calculated values of the explosive characteristics of azidobenzofuroxans **4a,b** exceed the currently used Cl-14 (Table 5).

## 3. Materials and Methods

General: The IR spectra were recorded on a Bruker Fourier spectrometer ALPHA (Bruker GmbH, Bremen, Germany) in the range 400–4000 cm^−1^. The ^1^H- and ^13^C-NMR spectra were recorded on a Bruker AVANCE 400 spectrometer (Bruker BioSpin, Rheinstetten, Germany) operating at 400 MHz (for ^1^H NMR) and 101 MHz (for ^13^C NMR). Chemical shifts were measured in δ (ppm) with reference to the solvent (δ = 2.06 ppm and 28.94 ppm for (CD_3_)_2_CO for ^1^H and ^13^C NMR, respectively). Elemental analysis was performed on a CHNS-O Elemental Analyser EuroEA3028-HT-OM (EuroVector S.p.A., Milan, Italy) with an accuracy of ±0.4%. The melting points were determined in glass capillaries on a Stuart SMP 10 instrument (Keison Products, Chelmsford, UK). Differential scanning calorimetry was performed on a NETZSCH DSC 204 F1 device (NETZSCH-Gerätebau GmbH, Selb, Germany) with a heating rate of 7 °C/min.

### 3.1. 7-(1,3-Diazido-2-(azidomethyl)propan-2-ylamino)-4,6-dinitrobenzofuroxan (***4a***) 

To a solution of 7-chloro-4,6-dinitrobenzofuroxan 2a (1.3 g, 0.005 mol) in CHCl_3_ (10 mL) at room temperature was added a 1,3-diazido-2-(azidomethyl)propan-2-amine 3 (1.96 g, 0.01 mol). The reaction was carried out at room temperature and under magnetic stirring, and the conversion was monitored through TLC analysis (eluent: toluene/ethyl acetate, 2/1). After 24 h the crude mixture was precipitated in hexane (20 mL), the obtained solid was filtered off, washed with cold water (100 mL), diethyl ether (20 mL) and dried under vacuum (0.06 mm Hg) at 40 °C temperature to constant weight. Yield 1.51 g (72%). Orange powder, m.p. = 104−105 °C. IR spectrum, ν, cm^−1^: 1550 (NO_2_), 1633 (furoxan ring), 2102 (N_3_), 3081 (H_bf_). ^1^H NMR (400 MHz, [D_6_]acetone, 25 °C) spectrum, δ, ppm: 10.76 (s, 1H, NH), 8.89 (s, 1H, H _bf_), 4.29 (s, 6H, 3CH_2_). ^13^C NMR ([D_6_]acetone) spectrum, δ, ppm: 148.15, 143.18, 126.89, 126.49, 123.32, 107.01, 64.01, 52.77. Anal. calcd (%) for C_10_H_8_N_14_O_6_. C 28.58; H 1.92; N 46.66. Found: 28.62; H 1.98; N 46.64.

### 3.2. 5,7-Bis(1,3-diazido-2-(azidomethyl)propan-2-ylamino)-4,6-dinitrobenzofuroxan (***4b***)

Was prepared analogously to compound **4a** from 0.005 mol of 4,6-dinitro-5,7-dichlorobenzofuroxan and 0.02 mol of 1,3-diazido-2-(azidomethyl)propan-2-amine. Yield 80%. Orange oil. IR spectrum, ν, cm^−1^: 1548 (NO_2_ asymm.), 1635 (furoxan ring), 2096 (N_3_). ^1^H NMR (400 MHz, [D_6_]acetone, 25 °C) spectrum, δ, ppm: 9.47 (2H, s, 2NH), 4.21 (12H, s, 6CH_2_). ^13^C NMR ([D_6_]acetone) spectrum, δ, ppm: 147.21, 143.09, 139.70, 132.06, 128.05, 106.76, 63.64, 52.66. Anal. calcd (%) for C_14_H_14_N_24_O_6_: C 27.37; H 2.30; N 54.71; Found: C 27.33; H 2.34; N 54.75.

### 3.3. Quantum Chemical Calculations

Calculations were performed with the Gaussian16 (Revision B.01) suite of programs [33]. The molecular structures of the investigated compounds were fully optimized using B3LYP hybrid functional [34,35] with 6-311++G (d,p) basis set with polarization and diffuse functions. The researched molecules were treated like molecules with closed electron shells, so the quantum-chemical calculations were carried out in singlet configuration. For all researched compounds, geometry optimization of structures was performed without symmetry constraints. The standard keywords in the Gaussian package were used in optimization processes. The tests of the stability of wave functions were carried out. To ensure the calculated structures of reagents and products were indeed minima, vibrational analyses were performed using the same methods and were proved by all positive eigenvalues of Hessian matrix.

### 3.4. TG/DSC Analysis

The thermogravimetry/differential scanning calorimetry analysis is performed using a NETZSCH STA449-F3 TG/DSC instrument. All researched samples (~1.6–7.4 mg) were placed in Al crucible with a perforated lid and heated from −75 to 350 °C together with an empty crucible as the reference. The measurements were carried out at a heating rate of 7 K/min in an argon flow of 50 mL/min.

## 4. Conclusions

New derivatives of benzofuroxan containing one or two triazidoisobutyl fragments were synthesized for the first time. A simple method was developed for their preparation with good yields under mild conditions. The obtained calculated data showed that the claimed azidobenzofuroxans in many thermodynamic parameters, such as potential, heat of combustion, as well as the enthalpy of formation, are superior to the compound CL-14: the potential is higher by 682 kJ/kg for **4a** and by 960 kJ/kg for **4b**, the value of heat combustion exceeds the specified indicator of CL-14 by 1025 kJ/kg for **4a** and 1209 kJ/kg for **4b**, the enthalpy of formation of the claimed compounds is 8–10 times higher than CL-14, which makes them promising components for obtaining high-energy compounds of a new generation. The raising of the potential (PP) of the compounds **4a** and **4b** calculated using REAL is in agreement with the results of quantum-chemical calculations and DSC measurement showing the increase of the enthalpies of formation and decomposition, respectively, of these energy-saturated products.

## Figures and Tables

**Table 1:** Melting temperatures (T_m_), gas-phase enthalpies of reaction (ΔH_r_(g)), gas-phase enthalpies of formation (ΔH_f_(g)), enthalpies of sublimation according to Trouton’s rule (ΔH_sub_), and solid-state enthalpy of formation (ΔH_f_).

Comp.	T_m_, K	ΔH_r_(g), kJ/kg	ΔH_f_(g), kJ/kg	ΔH_sub_, kJ/kg	ΔH_f_^a^, kJ/kg	ΔH_f_^b^, kJ/kg
4a	377	112.6	2825.8	168.7	2657.1	2611.7
4b	435	80.4	3499.1	133.1	3365.9	3279.8

^a^ according to method of isodesmic reactions; ^b^ according to general Equation (2) from [39].

**Table 2:** Results of the DSC study of compounds **4a,b**.

Compound	T_g_, °C	T_m.p._, °C	T_onset_, °C	T_d1_, °C	T_d2_, °C	T_d3_, °C	T_f.p._, °C
4a	−54.8	104.3 (43.4)	176.5	205.4 (337.2)	250.6 (10.5)	-	-
4b	−57.9 (−45.3) ^a^	-	145.6	162.9 (306.0)	228.1 (2.5)	248.2 (7.2)	-
1c (ADNBF) ^b^	-	-	-	-	-	-	279.6
1e (CL-14) ^b^	-	-	-	289.0	-	-	

^a^ the glass transition (or devitrification) temperature was determined by heating the sample from −70 °C to +350 °C; ^b^ data for ADNBF and CL-14 are taken from [42,43], respectively. T_g_—glass transition temperature; T_m.p._—melting temperature (in parentheses—enthalpies values, J/g); T_onset_—decomposition onset temperature; T_d_- decomposition temperature (in parentheses—enthalpies values, J/g); T_f.p._—deflagration point.

**Table 3:** Thermodynamic parameters calculation using REAL version 3.0.

Compound	DADNBF (CL-14)	4a	4b
Sum. formula	C_6_H_4_N_6_O_6_	C_10_H_8_N_14_O_6_	C_14_H_14_N_24_O_6_
Mol. mass [g/mol]	256.1	420.26	614.42
ρ [g/cm^3^]	1.942 ^a^ 1.910 ^b^	1.654 ^c^	solid oil
ΔH°_f_ [kJ/kg]	337.05 ^d^	2640.03 ^e^	3414.6 ^e^
OB Ω [%]	−49.97	−68.53	−75.52
T [K]	2992.97	3121.71	3096.78
µ [g/mol]	23.69	23.27	21.88
C_p_ [kJ/(kg∙K)]	1.61769	1.76853	1.87110
C_p_/C_v_	1.27250	1.23038	1.21592
C_ov_ [dm^3^/kg]	1.179	1.174	1.177
FP [kJ/kg]	1050.24	1040.91	1035.69
PP [kJ/kg]	3804.88	4487.11	4765.42
Q *^f^ [kJ/kg]	3688.209	4713.065	4897.836

^a^ [45]; ^b^ [46]; ^c^ gas pycnometer (21.5 °C); ^d^ [43]; ^e^ Calculated via Gaussian 16; ^f^ Q * is the heat of combustion at a “frozen” equilibria temperature of 1500 K (below which the combustion products do not undergo any chemical interactions); OB—oxygen balance; T—the temperature of the combustion products; µ—average molecular weight of combustion products; C_ov_—covolume; FP—specific energy (force); PP—potential.

**Table 4:** The composition of the reaction products (mol%) obtained using REAL version 3.0.

Compound	CL-14	4a	4b	Comp.	CL-14	4a	4b
C_2_H_2_	0.05905	1.22774	1.37763	NH_3_	0.06708	0.07333	0.10625
C_2_N_2_	-	0.05202	0.05549	CH_4_	0.15837	0.53265	0.78604
C_2_H_3_	-	0.03044	0.03723	H	0.11406	0.16742	0.17056
C_3_H	-	0.05927	0.05502	NCN	-	0.029	0.03091
CH_3_	-	0.07854	0.09924	HCN	1.1381	6.35595	7.26152
C_3_HN	-	1.84419	2.00062	HNCO	0.0239	-	-
C_4_H_2_	-	0.30659	0.32669	CO	53.47079	35.3204	24.07324
C_2_H_4_	-	0.07693	0.10976	H_2_	16.23264	16.26704	19.50845
CO_2_	0.45169	0.04108	-	N_2_	27.11562	37.27977	43.74823
H_2_O	1.07306	0.15374	0.13094				

**Table 5:** Calculated values of the explosive characteristics of azidobenzofuroxans **4a,b** and Cl-14.

Compound	V ^a^, L/kg	Q_v_^a^, kJ/kg	T_d_^a^, K	D, m/s	
				According to G.A. Avakyan ^a^ at ρ = 1.6	By Composition and Structure (According V.I. Pepekin) ^b^
CL-14 (IIb)	788	4031.86	3163.10	7441	8030
4a	693	5554.99	3581.71	8012	8036
4b	693	5866.77	3550.13	8220	8158

^a^ [47]; ^b^ [48]; V—volume of explosion gases; Q_v_—specific heat of explosion; T_d_—temperature of explosion; D—detonation velocity.

## Data Availability

The data presented in this study are contained within the article or are available upon request from the corresponding author Elena Chugunova.
